# Discriminative and Distinct Phenotyping by Constrained Tensor Factorization

**DOI:** 10.1038/s41598-017-01139-y

**Published:** 2017-04-25

**Authors:** Yejin Kim, Robert El-Kareh, Jimeng Sun, Hwanjo Yu, Xiaoqian Jiang

**Affiliations:** 1 Department of Creative IT Engineering, Pohang University of Science and Technology, Pohang, Korea; 2 Department of Biomedical Informatics, UC San Diego, La Jolla, CA United States; 3 School of Computational Science and Engineering, Georgia Institute of Technology, Atlanta, GA United States; 4 Department of Computer Science and Engineering, Pohang University of Science and Technology, Pohang, Korea

## Abstract

Adoption of Electronic Health Record (EHR) systems has led to collection of massive healthcare data, which creates oppor- tunities and challenges to study them. Computational phenotyping offers a promising way to convert the sparse and complex data into meaningful concepts that are interpretable to healthcare givers to make use of them. We propose a novel su- pervised nonnegative tensor factorization methodology that derives discriminative and distinct phenotypes. We represented co-occurrence of diagnoses and prescriptions in EHRs as a third-order tensor, and decomposed it using the CP algorithm. We evaluated discriminative power of our models with an Intensive Care Unit database (MIMIC-III) and demonstrated superior performance than state-of-the-art ICU mortality calculators (e.g., APACHE II, SAPS II). Example of the resulted phenotypes are sepsis with acute kidney injury, cardiac surgery, anemia, respiratory failure, heart failure, cardiac arrest, metastatic cancer (requiring ICU), end-stage dementia (requiring ICU and transitioned to comfort-care), intraabdominal conditions, and alcohol abuse/withdrawal.

## Introduction

A phenotype is an outward physical manifestation of a genotype. Investigating the association between phenotypes and genotypes has been a principal genetic research goal^1^. Electronic health records (EHRs) are increasingly used to identify phenotypes because EHRs encompass several aspects of patient information such as diagnoses, medication, laboratory results, and narrative reports. Given the importance of these efforts, collaborative groups have been created to develop and share phenotypes obtained from EHRs, such as the Electronic Medical Records and Genomics (eMERGE) Network^2^ and the Observational Medical Outcomes Partnership^3^. Two of the main obstacles to generate phenotypes are the needs for substantial time and domain expert knowledge^4, 5^. Furthermore, phenotypes created using clinical judgement^6, 7^ or healthcare guidelines^5, 8^ in one institution often cannot be easily ported to the other institutions, reducing generalizability and leading to unstandardized phenotype definitions^9^.

Consequently, phenotyping based on machine learning has been proposed to facilitate extraction of meaningful phenotypes automatically from EHRs without human supervision through a process called computational phenotyping. The most widely used approach is unsupervised feature extraction that derives meaningful and interpretable characteristics without supervision on data label. Frequent pattern mining defines phenotypes as a pattern that is frequently observed set of ordered items from sequential numerical data such as laboratory^10, 11^. A natural language processing technique extracts frequent terms from clinical narrative notes and defines phenotypes as a set of relevant and frequent terms^12–14^. These frequent set mining methods are useful but unable to learn underlying latent characteristics. Deep learning methods such as autoencoders or skip-grams represent patient as a vector^15–17^, but it is hard to derive understandable latent concepts due to the nonlinear combinations of multiple layers.

Recently, dimensionality reduction phenotyping methods have been introduced to handle sparse and noisy data from EHRs’ large and heterogeneous features. These methods represent phenotypes as latent medical concepts^18^. That is, the phenotypes are defined as a probabilistic membership to medical components, and patients also have a probabilistic membership to the phenotypes. For example, Bayesian finite mixture modeling discovers Parkinson’s disease phenotypes as latent subgroups^19^. Another dimensionality reduction technique, matrix factorization, decomposes time-series matrix data from EHRs into latent medical concepts^20–22^. Most recently, nonnegative tensor factorization (NTF) is becoming particularly popular due to its ability to capture high dimensional data. It generates latent medical concepts using interaction between components from multiple information source^23–27^. Ho *et al*. first introduce NTF for phenotyping^23, 24^. They define phenotypes as sets of co-occurring diagnoses and prescrptions, and obtain the phenotypes from latent representation of the co-occurrence. They use Kullback-Leibler divergence to decompose the observed co-occurrences that follow Poisson distribution based on CP decomposition. Ho *et al*. also incorporate sparsity constraints by setting thresholds for negligibly small values. Wang *et al*. enforce orthogonality constraints on NTF to derive less overlapping phenotypes^25^. Another NTF based on Tucker decomposition discovers (high-order) feature subgroups as decomposing the tensor into a core tensor multiplied by orthogonal factor matrices for each mode. It uses the core tensor to encode interactions among elements in each mode^26, 28^.

One of important characteristics that phenotypes should have is to be discriminative to a certain clinical outcome of interest such as mortality, readmission, cost, *et al*. So far, however, there has been little consideration about discriminative phenotypes associated with certain clinical outcomes. The discriminative phenotypes can be beneficial to clinicians because they can directly apply the phenotypes to their daily practice to improve the clinical outcome of interest. For example, clinicians can use our phenotype to evaluate patients’ risk of hospital death like APACHE II or SAPS score does, and improve resource allocation and quality-of-care in ICUs. Membership to the several different phenotypes can provide an insight on the situation of a patient beyond a single score. In addition, another crucial characteristic for phenotypes is to be distinct from each other, because otherwise clinicians cannot interpret and use the phenotypes easily. For example, let us say a patient suffers from hypertension and diabetes. To represent the patient, we can use a mixture of two phenotypes. We prefer Phenotype 1 = {hypertension, ACE inhibitors}, Phenotype 2 = {diabetes, insulin} to Phenotype 1 = {hypertension, ACE inhibitors, insulin}, Phenotype 2 = {diabetes}, because the former is more distinct and meaningful than the latter. Yet another critical concern about phenotypes is the compactness. Generally speaking, compact representation is more preferable than the lengthy one to end users if both have the same discrimination power and distinctness.

This paper proposes a new tensor factorization methodology for generating discriminative and distinct phenotypes. We defined phenotypes as the sets of co-occurring diagnoses and prescriptions. We used a tensor to represent diagnosis and prescription information from EHRs, and decomposed the tensor into latent medical concepts (i.e., phenotypes). To discriminate a high-risk group (high mortality), we incorporated the estimated probability of mortality from logistic regression during the decomposition process. We also found cluster structures of diagnoses and prescriptions using contextual similarity between the components, and absorbed the cluster structure into the tensor decomposition process.

## Methods

**Figure 1:**
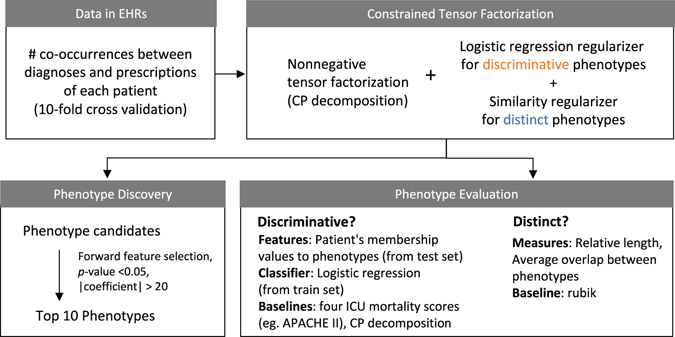
Workflow of our phenotyping method. We constructed a tensor using the number of co-occurrences between diagnoses and prescriptions of each patient in EHRs. We then decomposed the tensor using the proposed constrained tensor factorization that incorporates regularizers for discriminative and distinct phenotypes. We defined phenotype as a set of co-occurring diagnoses and prescriptions, which can be inferred using decomposed tensors, and evaluated their discriminative and distinct power. We also selected top 10 representative phenotypes and presented its meaning and usefulness.

We first describe a computational phenotyping method that we developed (Fig. 1) and experiment design.

### Phenotyping based on tensor factorization

**Figure 2:**
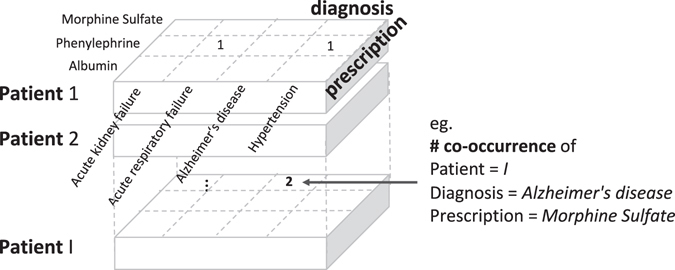
Constructing tensor from EHRs. We built a third-order tensor ${\mathscr{O}}$ with co-occurrences of patients, diagnoses, and prescriptions from EHRs. Patient *I* is diagnosed with *Alzheimer*’*s disease* and is ordered *morphine sulfate* twice.

We built a third-order tensor ${\mathscr{O}}$ with co-occurrences of patients, diagnoses, and prescriptions from intensive care unit (ICU) EHRs. Detailed tensor construction can be found in Supplementary methods. The co-occurrence is a natural representation of interactions between many diagnoses and prescriptions. We only focused on diagnosis and prescription data as previous phenotyping definition^29–31^, but we can extend the tensor to a high order (>3) to utilize additional data such as laboratory results and procedures. Specifically, we first built a matrix for individual patient to represent association between prescription and diagnosis. For example, let us say patient 1 is diagnosed with *acute respiratory failure* and *hypertension*, and is ordered the medicine *phenylephrine* during his or her admission. Then, each co-occurrence of *acute respiratory failure* and *phenylephrine*, and *hypertension* and *phenylephrine* is one, respectively (Fig. 2). Again, let us say patient *I* is diagnosed with *Alzheimer*’*s disease* and is ordered medicine *morphine sulfate* twice. Then, the co-occurrence of *Alzheimer*’*s disease* and *morphine sulfate* is 2. We collected all the matrices from all the patients and built the third-order observed tensor ${\mathscr{O}}$. Entries at (*i*, *j*, *k*) of the tensor (i.e., ${\mathscr{O}}$_*i*,*j*,*k*_) is the number of co-occurrence of diagnosis *j* and prescription *k* for patient *i*.

**Figure 3:**
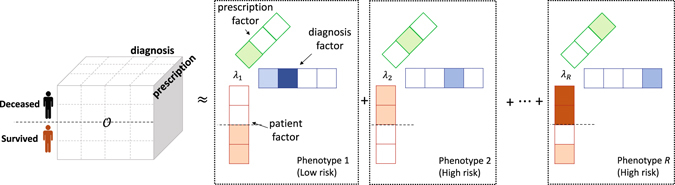
Phenotyping by tensor factorization. Dark shade, light shade, and no shade represents high membership, low membership, and zero membership to the phenotype, respectively. Patients who died have high membership to *Phenotype 2* and *Phenotype R*.

To decompose the tensor, we used CP algorithm^32, 33^; detailed description of CP can be found in Supplementary methods. Recently, phenotyping based on Tucker model has been proposed^26, 28^. It provides a more flexible modeling than does CP by allowing subgroups in each mode, but CP has an advantage in that it is computationally cheap and extendable by imposing regularizers. Using CP model, the third-order tensor ${\mathscr{O}}$ was decomposed into three factor matrices: **A** for patient mode, **B** for diagnosis mode, and **C** for prescription mode (Fig. 3). A phenotype consisted of diagnoses and prescriptions, and patients were involved in each phenotype. That is, the *r* th phenotype consisted of *J* diagnoses and *K* prescriptions with membership values that describe how much the diagnoses and prescriptions are involved and contribute to the *r* th phenotype. The membership values were normalized values between 0 and 1, and stored in the normalized vectors ${\overline{{\bf{B}}}}_{:r}$ and ${\overline{{\bf{C}}}}_{:r}$, respectively. Meanwhile, patients were involved in the *R* phenotypes with membership values that represent how much the patient has the characteristic of the phenotypes. The membership values of patients were also normalized values between 0 and 1, and stored in the normalized vector ${\overline{{\bf{A}}}}_{:r}$. Ability of *r* th phenotype that can capture and describe the data was stored in ${\lambda }_{r}=||{{\bf{A}}}_{:r}{||}_{F}||{{\bf{B}}}_{:r}{||}_{F}||{{\bf{C}}}_{:r}{||}_{F}$, because large values in **A**_:*r*_, **B**_:*r*_, and **C**_:*r*_ means that the *r* th phenotype describes large portion of co-occurrence values in ${\mathscr{O}}$. So, conversely, a phenotype with highly co-occurring diagnosis and prescription may have large *λ*_*r*_.

For example, ICU survived patients (half of total patients) have *Phenotype 1* in Fig. 3, which consists of the first two elements of diagnosis mode and the first one element of prescription mode. The second diagnosis element has higher membership to the *Phenotype 1* than the first element does. The patients who died in ICU have *Phenotype 2*, which consists of the third diagnosis and the second prescription. Similarly, the deceased patients and a few patients who survived have *Phenotype R*, which consists of the fourth diagnosis and the third prescription. Note that in this example, elements in a phenotype are not overlapped with elements in other phenotypes; thus, we can interpret the phenotype easily. Also, note that phenotypes for the deceased patients and the patients who survived are separated so that we can easily determine which phenotypes are more associated with mortality; consequently, we can further use the phenotypes to evaluate the risk of patients according to the membership to the phenotypes. We introduced two regularizations to make the phenotype discriminative and distinct in the following sections.

### Supervised phenotyping for discriminative power

We proposed a supervised approach to encourage the phenotypes separated according to mortality by adding a logistic regression regularization. In the previous section, patients had the membership values to the phenotypes. We used the membership as a feature vector to express patients, and used the feature vector to predict mortality. As a previous work on graph-based phenotyping method^21^, we added a regularization for supervised term. Let us say *y*_*i*_ is a binary indicator of mortality, i.e., *y*_*i*_ = 1 if *i* th patient dies during hospital admission and *y*_*i*_ = −1 otherwise. The *i* th patient in training set *L* (*i* ∈ *L*) was represented as the membership values to the phenotypes, **A**_*i*:_, which is the *i* th row vector of **A**. Given logistic regression parameters *θ*, a probability of *i* th patient’s mortality to be *y*_*i*_ is

1 $$P({{\bf{A}}}_{i:},{y}_{i}|\theta )=\frac{1}{1+\exp (-{y}_{i}{\delta }_{i})}$$

where *δ*_*i*_ = [**A**_*i*:_, 1] · *θ*. We then maximized the log-probability, or minimize the negative log-probability,

2 $${\rm{\min }}-\mathrm{log}\,P({{\bf{A}}}_{i:},{y}_{i}|\theta \mathrm{).}$$

Thus, the objective function for updating each row **A**_*i*:_ is

3 $$f({{\bf{A}}}_{i:})=||{{\bf{A}}}_{i:}{({\bf{C}}\odot {\bf{B}})}^{T}-{{\bf{O}}}_{\mathrm{(1)}i:}{||}_{F}^{2}-\omega \,\mathrm{log}\,P({{\bf{A}}}_{i:},{y}_{i}|\theta \mathrm{).}$$

with a weighting constant *ω* ($\odot$ refers to Khatri-Rao product). Note that this objective function is with respect to row **A**_*i*:_ not the whole patient factor matrix **A**. Gradient of *f*(**A**_*i*:_) is

4 $$\nabla f({{\bf{A}}}_{i:})=2{{\bf{A}}}_{i:}{({\bf{C}}\odot {\bf{B}})}^{T}({\bf{C}}\odot {\bf{B}})-2{{\bf{O}}}_{\mathrm{(1)}i:}({\bf{C}}\odot {\bf{B}})-\omega {y}_{i}\frac{1}{1+\exp ({y}_{i}{\delta }_{i})}{\theta }^{T}$$

and hessian of *f*(**A**_*i*:_) is

5 $${\nabla }^{2}f({{\bf{A}}}_{i:})=\mathrm{2(}{\bf{C}}\odot {\bf{B}}{)}^{T}({\bf{C}}\odot {\bf{B}})+\omega \frac{1}{2+\exp ({y}_{i}{\delta }_{i})+\exp (-{y}_{i}{\delta }_{i})}\theta {\theta }^{T}\mathrm{.}$$

Using Newton’s gradient descent method, if *i* ∈ *L*, we update **A**_*i*:_ as

6 $${{\bf{A}}}_{i:}=max(0,\,{{\bf{A}}}_{i:}-{{\rm{\nabla }}}^{2}f{({{\bf{A}}}_{i:})}^{-1}{\rm{\nabla }}f({{\bf{A}}}_{i:})).$$

If *i* ∉ *L*, we update **A**_*i*:_ as Eq. (6) with *ω* = 0, which is a traditional CP decomposition without any regularization. Time complexity of Eq. (6) is bounded by *O*(*JKR* ^2^) for *i* ∈ *L*; total time complexity to update **A** is bounded by *O*(*IJKR* ^2^) (Table S1). The supervised term had negligible effects on the total time complexity. This updating rule can be linearly scaled up to the size of **A**. Updating the logistic regression parameters *θ* followed a typical logistic regression modeling method. We added a ridge penalty to shrink the size of *θ* and avoid overfitting (*c* is a weighting constant)^34^ as

7 $${\rm{\min }}-\mathrm{log}\,P({{\bf{A}}}_{i:},{y}_{i}|\theta )+c||\theta {||}^{2}\mathrm{.}$$

### Similarity-based phenotyping for distinct power

To derive distinct phenotypes with less overlapping with each other, we made phenotypes only consist of similar elements. We first derived components’ similarities from contexts in EHRs, used the similarities to infer cluster structures, and let phenotypes reflect the cluster structures.

### Deriving contextual similarity

We derived contextual similarities from EHRs. Farhan *et al*. generate a vector representation of medical events (or elements in phenotype)^17^. Based on this work, we generated sequences that consist of diagnoses and prescriptions from EHRs in time order (Table 1). We applied Word2Vec, a two-layer neural network for natural language processing for numerical representation of discrete words^35^. We input the time-ordered EHRs sequences into Word2Vec and derived a set of vectors for each diagnosis or prescription. After several trials, we set cardinality of the vector as 500 and window size of the sequence (i.e., the number of diagnoses or prescriptions in a sequence to consider them contextually similar) as 30. We found that, as the cardinality increases, distribution of the pairwise similarities spreads widely (i.e., many similarity values are close to −1 or 1 other than 0), but computation time also increases rapidly. We also observed that most of the pairwise similarities become close to 0 as the window size decreases, and close to 1 as the window size increases.

**Table 1:** Examples of time-ordered EHRs sequences.

Lorazepam → Acetaminophen → Piperacillin-Tazobactam → Ventricular fibrillation
Diltiazem → Pneumococcal Vac Polyvalent → Anemia → Chronic obst asthma
Pantoprazole Sodium → Acetaminophen
Oxycodone → Heparin Flush → Severe sepsis

Each sequence consists of formulary drug codes (prescription) and ICD-9 codes (diagnosis), and is used in Word2Vec to derive pairwise similarities.

We then computed cosine similarities between the vector representation of elements, and derived a pairwise similarity matrix (either *J* × *J* matrix **S** ^*B*^ for diagnosis or *K* × *K* matrix **S** ^*C*^ for prescription). For example, let us say the *j*_1_ th and *j*_2_ th diagnoses in our dataset refer to *atrial fibrillation* and *congestive heart failure*, respectively. The vector representation is *atrial fibrillation* = (0.1, 0.6, 0.2, 0.1) and *congestive heart failure* = (0.3, 0.7, 0.1, 0.2). The similarity between them is stored at (*j*_1_, *j*_2_)-entry of **S** ^*B*^, and the value is ${{\bf{S}}}_{{j}_{1},{j}_{2}}^{B}=\frac{0.1\times 0.3+0.6\times 0.7+0.2\times 0.1+0.1\times 0.2}{\sqrt{0.42}\sqrt{0.63}}\approx 0.95$.

We made **S** sparse for efficiency by ignoring trivial values. Many similarities were close to zero, and their small variance did not provide useful information. Similarities less than zero refer to dissimilarity, which was not the focus of this work. Considering all the less useful similarity values can increase computational overhead. We only used the highest *l* similarities value for each element, and consider the others as 0. We choose $l=\lfloor {\mathrm{log}}_{2}J\rfloor$ for diagnosis and $\lfloor {{\rm{l}}{\rm{o}}{\rm{g}}}_{2}K\rfloor (l > 0)$ for prescription according to previous works^36, 37^.

We converted **S** into a normalized-cut similarity matrix^38^. Incorporating the normalized cut similarity helped our problem to increase both the total dissimilarity between the different phenotypes and the total similarity within the phenotypes, thus avoid overlapping between the phenotypes. Converting to the normalized cut similarity matrix is

8 $${\bf{S}}\leftarrow {{\bf{D}}}^{-\frac{1}{2}}{\bf{S}}{{\bf{D}}}^{-\frac{1}{2}}$$

where **D** is a diagonal matrix of ${\bf{D}}=diag({d}_{1},\ldots ,{d}_{J}),{d}_{j}={\sum }_{l=1}^{J}{{\bf{S}}}_{jl}$.

### Incorporating cluster structure

With the similarity matrix, we inferred a cluster structure from the similarity and incorporated it to our NTF optimization. The cluster structure contained information on which elements should be in the same phenotype together. We introduced a regularization term for the spectral clustering. We increased the sum of pairwise similarity within a phenotype. Because how much the elements are involved in each phenotype is different, the pairwise similarity was weighted by the two elements’ membership values to the phenotype. That is, in terms of diagnosis similarity matrix **S** ^**B**^, the sum of weighted pairwise similarity within a phenotype *r* is

9 $$\sum _{{j}_{1}=1}^{J}\,\sum _{{j}_{2}=1}^{J}{{\bf{B}}}_{{j}_{1},r}{{\bf{B}}}_{{j}_{2},r}{{{\bf{S}}}^{{\bf{B}}}}_{{j}_{1},{j}_{2}},$$

and the sum of all the similarity in Eq. (9) throughout the *R* phenotypes is

10 $$\begin{array}{l}\sum _{r=1}^{R}\,\sum _{{j}_{1}=1}^{J}\,\sum _{{j}_{2}=1}^{J}{{\bf{B}}}_{{j}_{1},r}{{\bf{B}}}_{{j}_{2},r}{{{\bf{S}}}^{{\bf{B}}}}_{{j}_{1},{j}_{2}}=\sum _{r=1}^{R}{\rm{Tr}}({{\bf{B}}}_{:r}^{T}{{\bf{S}}}^{{\bf{B}}}{{\bf{B}}}_{:r})={\rm{Tr}}({{\bf{B}}}^{T}{{\bf{S}}}^{{\bf{B}}}{\bf{B}}\mathrm{).}\end{array}$$

Here, Tr(**B** ^*T*^ **S** ^**B**^ **B**) is the objective of spectral clustering in which **B** represent the clustering assignment of each element^37^. Consequently, the phenotypes preserved the spectral clustering structure by incorporating sum of weighted similarity. Meanwhile, *Tr*(**B** ^*T*^ **S** ^**B**^ **B**) is also equivalent to symmetric nonnegative matrix factorization of similarity matrix **S** ^**B**^ ^36, 39^, i.e.,

11 $${\rm{\max }}\,{\rm{Tr}}({{\bf{B}}}^{T}{{\bf{S}}}^{{\bf{B}}}{\bf{B}})\leftrightarrow \,{\rm{\min }}\,{\Vert {{\bf{S}}}^{{\bf{B}}}-{\bf{B}}{{\bf{B}}}^{T}\Vert }^{2}$$

because

12 $$\begin{array}{rcl}{\rm{\max }}\,{\rm{Tr}}({{\bf{B}}}^{T}{{\bf{S}}}^{{\bf{B}}}{\bf{B}})\leftrightarrow \,{\rm{\min }}\,-2{\rm{Tr}}({{\bf{B}}}^{T}{{\bf{S}}}^{{\bf{B}}}{\bf{B}}) & = & \min \,||{\bf{S}}{||}^{2}-2{\rm{Tr}}({{\bf{B}}}^{T}{{\bf{S}}}^{{\bf{B}}}{\bf{B}})+||{{\bf{B}}}^{T}{\bf{B}}{||}^{2}\\  & = & \min \,||{{\bf{S}}}^{{\bf{B}}}-{\bf{B}}{{\bf{B}}}^{T}{||}^{2}\end{array}$$

by relaxing a constraint on **B** ^*T*^ **B** = **I** ^39^. This transformation is beneficial because it helps phenotypes to be more orthogonal (or distinct) by retaining **B** ^*T*^ orthogonality approximately^39^. Thus, the objective function with the cluster structure is

13 $$g({\bf{B}})=||{\bf{B}}{({\bf{C}}\odot {\bf{A}})}^{T}-{{\bf{O}}}_{\mathrm{(2)}}{||}^{2}+\mu ||{{\bf{S}}}^{{\bf{B}}}-{\bf{B}}{{\bf{B}}}^{T}{||}^{2}$$

with a weighting constant *μ*. By incorporating $||{{\bf{S}}}^{{\bf{B}}}-{\bf{B}}{{\bf{B}}}^{T}{||}^{2}$, our phenotyping method can absorb the spectral clustering structure and improve the orthogonality at the same time. Although it is a fourth-order non-convex function and it is difficult to find a global optimum, it can converge to a stationary point^36^. To find an optimum value, we derived the gradient of *g*(**B**):

14 $$\nabla g({\bf{B}})=2{\bf{B}}{({\bf{C}}\odot {\bf{A}})}^{T}({\bf{C}}\odot {\bf{A}})-2{{\bf{O}}}_{\mathrm{(2)}}{\bf{C}}\odot {\bf{A}}+4\mu ({\bf{B}}{{\bf{B}}}^{T}-{{\bf{S}}}^{{\bf{B}}}){\bf{B}},$$

and hessian of *g*(**B**):

15 $$\begin{array}{rcl}{\nabla }^{2}g(vec({\bf{B}})) & = & \mathrm{2(}{\bf{C}}\odot {\bf{A}}{)}^{T}({\bf{C}}\odot {\bf{A}})\otimes {{\bf{I}}}_{J\times J}+4\mu \mathrm{(2}vec({\bf{B}})vec{({\bf{B}})}^{T}\\  &  & +\,vec{({\bf{B}})}^{T}vec({\bf{B}}){{\bf{I}}}_{JR\times JR}-{{\bf{I}}}_{R\times R}\otimes {{\bf{S}}}^{{\bf{B}}}),\end{array}$$

where a *vec*(**B**) of length *JR* is a vectorization of **B** by column i.e., $vec({\bf{B}})=[{{\bf{B}}}_{\mathrm{:1}}^{T},\ldots ,{{\bf{B}}}_{:R}^{T}{]}^{T}$, and $\otimes$ refers to Kronecker product. Using Newton’s gradient descent method, we updated **B** as

16 $$vec({\bf{B}})=\,\max (0,\,vec({\bf{B}})-{\nabla }^{2}g{(vec({\bf{B}}))}^{-1}\nabla g(vec({\bf{B}})))\mathrm{.}$$

Time complexity of Eq. (16) is bounded by *O*(*IJKR*) + *O*(*J* ^3^ *R* ^3^). The similarity term had negligible effects on the total time complexity (Table S2). The updating rule for **B** contained matrix inversion of ${\nabla }^{2}g(vec({\bf{B}}))\in {{\mathbb{R}}}^{JR\times JR}$, which may not be scaled up well with large *J*. In this case, we can use a constant learning rate instead of ${\nabla }^{2}g{(vec({\bf{B}}))}^{-1}$ although sacrificing converging rate.

Similarly, the factor matrix **C** for prescriptions followed the same update procedure. We repeated the updating procedures for the factor matrices **A**, **B** and **C** and logistic regression parameter *θ* until convergence. We assumed convergence when $||fi{t}_{old}-fit|| < 5\times {10}^{-4}$ where *fit* is defined as $fit=1-\frac{||{\mathscr{O}}-{\mathscr{X}}||}{||{\mathscr{O}}||}$, and *fit*_*old*_ is the *fit* of the previous iteration. After normalizing, we removed trivial values less than threshold *ε* because those values are too small for meaningful membership value and worsen the conciseness. We summarized the entire updating procedures in Algorithm 1.

**Table:** 

**Input**: $${\mathscr{O}},\omega ,\mu $$
1: Randomly initialize **A**, **B**, **C**.
2: **repeat**
3: $${{\bf{A}}}_{i:}=\,\max (\mathrm{0,}\,{{\bf{A}}}_{i:}-{\nabla }^{2}f{({{\bf{A}}}_{i:})}^{-1}\nabla f({{\bf{A}}}_{i:}))$$ for all *i*.
4: Update *θ* for logistic regression
5: $$vec({\bf{B}})=\,{\rm{\max }}(\mathrm{0,}\,vec({\bf{B}})-{\nabla }^{2}g{(vec({\bf{B}}))}^{-1}\nabla g(vec({\bf{B}})))$$.
6: $$vec({\bf{C}})=\,{\rm{\max }}(\mathrm{0,}\,vec({\bf{C}})-{\nabla }^{2}g{(vec({\bf{C}}))}^{-1}\nabla g(vec({\bf{C}})))$$.
7: **until** Converged
8: $${\overline{{\bf{A}}}}_{:r}\leftarrow \frac{{{\bf{A}}}_{:r}}{||{{\bf{A}}}_{:r}||}$$, $${\overline{{\bf{B}}}}_{:r}\leftarrow \frac{{{\bf{B}}}_{:r}}{||{{\bf{B}}}_{:r}||}$$, $${\overline{{\bf{C}}}}_{:r}\leftarrow \frac{{{\bf{C}}}_{:r}}{||{{\bf{C}}}_{:r}||}$$, $$\forall r$$
9: $${\overline{{\bf{A}}}}_{ir}\leftarrow 0\,{\rm{if}}\,{\overline{{\bf{A}}}}_{ir} < {10}^{-6}$$, $${\overline{{\bf{B}}}}_{jr}\leftarrow 0\,{\rm{if}}\,{\overline{{\bf{B}}}}_{jr} < {10}^{-3}$$, $${\overline{{\bf{C}}}}_{kr}\leftarrow 0\,{\rm{if}}\,{\overline{{\bf{C}}}}_{kr} < {10}^{-3}\,\forall i,j,k,r$$
10: **return** $${\mathscr{X}}={\sum }_{r=1}^{R}{\lambda }_{r}{\overline{{\bf{A}}}}_{:r}{\overline{{\bf{B}}}}_{:r}{\overline{{\bf{C}}}}_{:r}$$.

### Experiment design

#### Dataset and preprocessing

We used a large publicly available dataset MIMIC-III (Medical Information Mart for Intensive Care III)^40^. MIMIC-III contains comprehensive de-identified data on around 46,520 patients in critical care units of the Beth Israel Deaconess Medical Center between 2001 and 2012, and it includes information such as demographics, prescription, diagnosis ICD codes, and clinical outcomes such as mortality. We selected 10,028 patients, including all 5,014 patients who died during admission and a random sample of 5,014 of patients who survived. If a patient who survived had multiple admission histories, we used the first admission. We used 202 diagnosis ICD-9 codes that are appeared in the charts of at least 5% of the patients and 316 prescription codes that appeared in at least 10% of the patients. We excluded diagnosis ICD-9 ‘V’ or ‘E’ codes that describe supplementary factors for health status. We excluded trivial base type prescriptions such as 0.9% sodium chloride, 5% dextrose, and sterile water. Most nonzero co-occurrence values are one, and skewed right (Fig. S1). To prevent small-dosage frequent medicines from having high co-occurrences, we truncated the co-occurrence values to 1% percentile, 10 (Fig. S1).

#### Evaluation measures

We evaluated our proposed method in terms of discrimination and distinction. We measured the discrimination by the area under the receiver operating characteristic curve (AUC), sensitivity, and specificity. We measured distinction by a relative length of phenotype and an average overlap. An absolute length of *r* th phenotype is the number of nonzero in membership vector **B**_:*r*_ and **C**_:*r*_. The relative length of the phenotype is the absolute length divided by the maximum length *J* + *K*. We averaged the *R* relative lengths of phenotype. The average overlap^41^ measures the degree of overlapping between all phenotype pairs. It is defined as the average of cosine similarities between phenotype pairs:

17 $${\rm{Avg}}\,{\rm{Overlap}}=\frac{{\sum }_{{r}_{1}}^{R}{\sum }_{{r}_{2} > {r}_{1}}^{R}\{\cos ({{\bf{B}}}_{:{r}_{1}},{{\bf{B}}}_{:{r}_{2}})+\,\cos ({{\bf{C}}}_{:{r}_{1}},{{\bf{C}}}_{:{r}_{2}})\}}{R(R-\mathrm{1)}}\mathrm{.}$$

Setting *R* = 50, we repeatedly ran our models ten times for 10-fold cross validation. We used the training set to compute the regression parameter *θ* and the likelihood term in supervised phenotyping, and used the test set to measure the discrimination (Table S3). Because tensor factorization is not deterministic method, the factorized tensors are different in each trial; so, we computed mean and 95% confidence interval.

#### Baselines

We compared the discrimination and the distinction of our proposed methods with that of several baseline methods. The baselines are:

- APACHE II, SAPS II, OASIS, APS III score: Disease severity scores for predicting mortality in intensive care unit (for comparing discrimination only)^42–45^. These scores assess the severity of disease using variables from pre-existing conditions, physiological measurements, biochemical/hematological indices, and source of admission. The weighted sum of individual values produces the severity scores^46^.
- CP: Basic NTF model^47, 48^.
- Rubik: A state-of-the-art computational phenotyping method based on CP. Rubik generates a phenotype candidate using count of diagnoses and treatments. It incorporates the orthogonality between phenotypes to derive concise phenotypes^41^. We assume no existing knowledge term and bias term.
  Our proposed methods are:
  - The **supervised phenotyping** that incorporates the prediction term for discriminative phenotypes (*ω* ≠ 0, *μ* = 0).
  - The **similarity-based phenotyping** that incorporates the cluster structure term for distinct phenotypes (*ω* = 0, *μ* ≠ 0).
  - The final model that incorporates the **both** supervised and similarity-based approach (*ω* ≠ 0, *μ* ≠ 0).

When evaluating discrimination (AUC, sensitivity, specificity) of NTF-based models, we used the patient’s membership values (i.e., ${\overline{{\bf{A}}}}_{i:}$ of size 1 × *R*) as features to fit a binary logistic regression to predict mortality. Particularly, for the supervised model, we fitted a binary logistic regression (after normalization) other than *θ* that are used during updating procedures. To examine the performance of the supervised and similarity-based phenotyping respectively, we compared the discrimination of CP and the supervised phenotyping (regardless of similarity term), and also compared the distinction of Rubik and similarity-based phenotyping (regardless of supervised term). We then combined the supervised approach and similarity-based approach together to achieve both discrimination and distinction. The weighting constants *ω* and *μ* were selected as *ω* = 1 and *μ* = 1000 after several trials. Note that *ω* was comparably small because it sensitively applied to each row of **A** whereas *μ* applies to the *l*_2_ norm of the whole matrix **B** or **C**. We used a tensor Matlab Tensor Toolbox Version 2.5^49^ from Sandia National Laboratories to represent tensors and compute tensor operations.

## Results

We present the experimental evaluation and phenotypes derived from our method.

### Discriminative and distinction power comparison

We found that our methods outperformed other baselines in terms of discrimination and distinction. The supervised phenotyping method showed the highest AUC and sensitivity among the other methods including APACHE II and SAPS II (Table 2). The similarity-based phenotyping method showed the lowest relative length and average overlap among the other methods. Particularly when compared with Rubik^25^ that considers orthogonality for the distinction, the similarity-based method improved the distinction significantly (the relative length of 0.3934 vs 0.0714).

**Table 2:** Discriminative and distinction power comparison.

	RMSE	AUC	Sensitivity	Specificity	Rel. Length	Avg. overlap
APACHE II^42^	—	0.7364	0.6712	0.6728	—	—
SAPS II^43^	—	0.8129	0.7970	0.6720	—	—
OASIS^44^	—	0.7227	0.6253	0.7077	—	—
APS III^45^	—	0.7419	0.6861	0.6994	—	—
CP^32, 33^	2.2153 (±0.0015)	0.8469 (±0.0156)	0.8375 (±0.0391)	0.7342 (±0.0401)	0.6807 (±0.0047)	0.3777 (±0.0064)
Supervised	2.2152 ±(0.0016)	0.8568 (±0.0106)	0.8392 (±0.0377)	0.7518 (±0.0393)	0.6828 (±0.0019)	0.3787 (±0.0059)
Rubik^25^	2.5025 (±0.0003)	0.7779 (±0.0247)	0.7310 (±0.0304)	0.7242 (±0.0377)	0.3934 (±0.0102)	0.2806 (±0.0075)
Sim.-based	2.5069 (±0.0130)	0.7796 (±0.0204)	0.7615 (±0.0378)	0.7097 (±0.0473)	0.0714 (±0.0406)	0.0013 (±0.0014)
Supervised + Sim.-based	2.3014 (±0.0060)	0.8389 (±0.0199)	0.8223 (±0.0387)	0.7487 (±0.0409)	0.3958 (±0.0137)	0.1267 (±0.0100)

RMSE, discrimination (AUC, sensitivity, specificity) and distinction (Relative length, Average overlap) with 95% confidence interval of baselines and our proposed models when *R* = 50. CP = CP decomposition, Supervised = the supervised phenotyping for discriminative power, Sim.-based = the similarity-based phenotyping for distinct power, Supervised + Sim. -based = the final model that incorporates the both supervised and similarity-based phenotyping.

### Phenotypes

We presented the phenotypes that are derived from the similarity-based phenotyping method for maximum conciseness. After the tensor decomposition procedures with *R* = 50, we selected 25 phenotypes by forward feature selection^50^ to remove phenotypes that are redundant and not statistically significant for predicting mortality (Table 3). Among them, we reported ten representative phenotypes in which coefficients from the feature selection were large enough (absolute value of coefficient >20) to discriminate mortality (Table 4): sepsis with acute kidney injury, cardiac surgery, anemia, respiratory failure, heart failure, cardiac arrest, metastatic cancer (requiring ICU), end-stage dementia (requiring ICU – sepsis, aspiration, trauma – and transitioned to comfort care), intraabdominal conditions, and alcohol abuse/withdrawal.

**Table 3:** Logistic regression coefficient from feature selection, *p*-value, and prevalence.

Phenotype	Coefficient	*p*-value	*λ*	Prevalence
Intercept	−0.19	<0.001	—	—
1	28.47	<0.001	749	94.53
3: Sepsis with acute kidney injury	44.64	<0.001	96	45.24
4: Cardiac surgery	−138.00	<0.001	95	50.43
5: Anemia	−19.76	<0.001	58	36.81
6: Respiratory failure	88.87	<0.001	56	30.98
10: Heart failure	30.79	<0.001	39	27.19
11	15.13	<0.001	37	16.74
13	−15.23	<0.001	31	22.48
15	−7.74	0.02	30	19.02
16	8.69	<0.001	29	42.99
18: Cardiac arrest	47.08	<0.001	28	9.14
20	−11.49	<0.001	23	9.70
21	−5.54	0.02	22	18.46
23: Metastatic cancer requiring ICU	25.10	<0.001	20	12.29
24: End-stage dementia requiring ICU	34.46	<0.001	20	12.72
25	12.81	<0.001	18	15.08
28	−9.00	<0.001	17	10.23
29	10.78	<0.001	16	18.06
31	10.42	0.01	16	6.13
32: Intraabdominal conditions	−19.21	<0.001	15	4.84
33	−6.41	0.04	14	5.12
34: Alcohol abuse/withdrawal	−22.82	<0.001	13	12.57
41	−19.89	<0.001	10	16.23
46	13.54	<0.001	8	7.20
47	−9.78	<0.001	6	7.96

Ten representative phenotypes are 3: Sepsis with acute kidney injury, 4: Cardiac surgery, 5: Anemia, 6: Respiratory failure, 10: Heart failure, 18: Cardiac arrest, 23: Metastatic cancer requiring ICU, 24: End-stage dementia requiring ICU for comport care, 32: Intraabdominal conditions, 34: Alcohol abuse/withdrawal. *λ*_*r*_ = ||**A**_:*r*_||_*F*_||**B**_:*r*_||_*F*_||**C**_:*r*_||_*F*_ (for frequency). Prevalence = (the number of patients whose membership to the phenotype is non-zero/the total number of patients) × 100%.

**Table 4:** Ten representative phenotypes. Listed in order of frequency.

Sepsis with acute kidney injury		Cardiac surgery (CABG/valve replacements)	
Diagnosis	Prescription	Diagnosis	Prescription
Acute kidney failure NOS, Acute kidny fail - tubr necr, Acute respiratry failure, Severe sepsis, Septic shock, Septicemia NOS	Vancomycin, Ciprofloxacin, Piperacillin-Tazobactam, CefePIME, Linezolid, Meropenem, Miconazole Powder, Nystatin Oral Suspension, Alteplase, Fluconazole, Loperamide HCl	Hypertension NOS, Crnry athrscl natve vssl, Hyperlipidemia NEC/NOS, Atrial fibrillation, DMII wo cmp nt st uncntr, Pure hypercholesterolem, Surg compl-heart, Aortic valve disorder	Phenylephrine HCl, Neostigmine, Aspirin EC, Ketorolac, Oxycodone-Acetaminophen, Ranitidine, Milk of Magnesia, Furosemide, Ibuprofen, TraMADOL (Ultram)
**Anemia (variation in other diagnoses)**		**Respiratory failure**	
Anemia NOS, Ac posthemorrhag anemia, Chr blood loss anemia, Iron defic anemia NOS	Insulin, Metformin	Acute respiratry failure, Pulmonary insufficiency following trauma and surgery, Other pulmonary insuff, Acute & chronc resp fail	Albumin, PHENYLEPHrine, Dextrose 50%, Chlorhexidine Gluconate, Milrinone, Epinephrine
**Heart failure**		**Cardiac arrest**	
CHF NOS	Morphine Sulfate, Nitroprusside Sodium, Nitroglycerin, Aspirin EC, Sucralfate	Ventricular fibrillation, Cardiogenic shock, Parox ventric tachycard, Atriovent block complete, Cardiac arrest, AMI anterior wall - init	Acetaminophen IV, Fentanyl Citrate, Influenza Virus Vaccine, Morphine Sulfate, NORepinephrine, Glucagon, Readi-Cat 2, Midazolam, Omeprazole
**Metastatic cancer requiring ICU (cord compression, need for bronch, etc)**		**End-stage dementia requiring ICU (sepsis, aspiration, trauma) and transitioned to comfort care**	
Secondary malig neo bone, Secondary malig neo brain/spine, Secondary malig neo lung, Secondary malig neo liver, Neurohypophysis dis NEC	Propofol, Midazolam, Fentanyl Citrate, Dexmedetomidine HCl, Vecuronium Bromide	Alzheimer’s disease, Paralysis agitans, Dementia w/o behav dist, Mental disor NEC oth dis	Morphine Sulfate, Scopolamine Patch
**Intraabdominal conditions–alcoholic pancreatitis, gallstone pancreatitis, perforated ulcer, etc**		**Alcohol abuse/withdrawl**	
Paralytic ileus, Digestive system complications not elsewhere classified, Acute pancreatitis, Cholangitis	Captopril, Metoprolol Tartrate	Alcohol dep NEC/NOS-unspec, Alcohol withdrawal, Alcohol dep NEC/NOS-contin, Bipolar disorder NOS	Hydromorphone, Diphenhydramine HCl, Morphine Sulfate, Prochlorperazine

**Figure 4:**
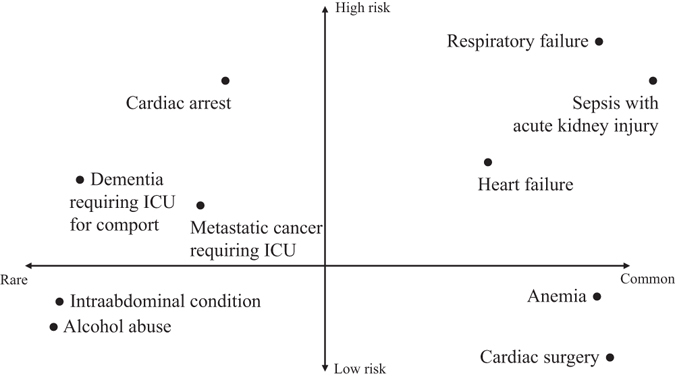
Phenotype maps. Phenotypes are positioned according to frequency and mortality risk.

We categorized the phenotypes into four groups according to frequency (common or rare) and risk (high or low). Common phenotypes were the top five with high *λ* and prevalence (and rare otherwise). High-risk (low-risk) phenotypes were ones with positive (negative) logistic regression coefficients (Table 3). As a result, common and high-risk phenotypes are sepsis with acute kidney injury, respiratory failure, and heart failure; rare but high-risk phenotypes are cardiac arrest, metastatic cancer requiring ICU, and end-stage dementia requiring ICU; common but low-risk phenotypes are anemia and cardiac surgery; and rare and low-risk phenotypes are intraabdominal conditions and alcohol abuse/withdrawal (Fig. 4).

To examine the risk of each phenotype in detail, we computed mortality of patients who were highly involved to each phenotype (Table 5). We observed that the mortality of patients who have high membership to phenotypes that are denoted as high-risk in Fig. 4 tends to increase to 1.

**Table 5:** Patient’s mortality distribution.

Phenotype	Membership									
	[0, 0.1)	[0.1, 0.2)	[0.2, 0.3)	[0.3, 0.4)	[0.4, 0.5)	[0.5, 0.6)	[0.6, 0.7)	[0.7, 0.8)	[0.8, 0.9)	[0.9, 1)
Sepsis with acute kidney injury	0.48	0.79	0.80	0.85	0.82	0.87	0.63	0.86	—	—
Cardiac surgery	0.58	0.39	0.25	0.18	0.08	0.05	0.04	0.04	0.04	0.05
Anemia	0.53	0.49	0.50	0.35	0.34	0.30	0.29	0.24	0.10	0.18
Respiratory failure	0.48	0.84	0.85	0.91	0.86	0.88	0.80	0.77	0.92	0.73
Heart failure	0.50	0.72	0.74	0.67	0.67	0.65	0.64	0.73	0.71	0.84
Cardiac arrest	0.51	0.83	0.76	0.84	0.85	0.91	1.00	0.83	0.88	1.00
Metastatic cancer requiring ICU	0.51	0.80	0.71	0.81	0.65	0.78	0.87	0.80	0.75	0.74
End-stage dementia requiring ICU	0.51	0.81	0.80	0.81	0.74	0.75	0.90	0.93	0.84	0.91
Intraabdominal conditions	0.52	0.52	0.39	0.45	0.38	0.33	0.17	0.27	—	—
Alcohol abuse/withdrawal	0.53	0.44	0.36	0.36	0.30	0.42	0.20	0.13	0.08	0.19

The distribution is computed as the number of patients who died/the total number of patients whose membership value is in the range. Empty values when the number of patients <10. Note that our dataset contained half patients who died and half patients who survived.

## Discussion

The objective of this study was to develop a phenotyping method that can generate discriminative and distinct phenotypes. As a result, we derived phenotypes that consist of interactions between related diagnoses and prescriptions, and patients had membership to each phenotype. The phenotypes from the supervised model were more discriminative than APACHE II, SAPS II scores and the phenotypes from CP model^32, 33^; the phenotypes from the similarity-based model were more distinct than the phenotypes from Rubik^25^. We also observed that the supervised phenotyping and the similarity-based phenotyping have an opposite effect on each other in terms of the discrimination and distinction. The distinct phenotypes from the similarity-based approach lost its discriminative power, and the discriminative phenotypes from the supervised approach lost distinction power. A possible explanation for this trade-off is that the similarity-based model tends to ignore less relevant elements in a phenotype to achieve the best distinction, although the “less relevant elements“ can contribute to increasing the discriminative power overall. However, the combined phenotypes from both approaches achieved the high discrimination and distinction at the same time (Table 2). When combining the supervised and the similarity-based phenotyping, the discrimination increased (with the AUC of 0.8389) compared to the similarity model (with the AUC of 0.7796), and distinction improved (with the relative length of 0.3958 and average overlap of 0.1267) compared to the supervised model (with the relative length of 0.6828 and average overlap of 0.3787).

We also described the most representative phenotypes: sepsis with acute kidney injury, cardiac surgery, anemia, respiratory failure, heart failure, cardiac arrest, metastatic cancer (requiring ICU), end-stage dementia (requiring ICU and transitioned to comfort care), intraabdominal conditions, and alcohol abuse/withdrawal. These conditions are fairly consistent with the list of conditions known to require ICU care in US hospitals^51^.

Our study also had some limitations. One limitation is that our approach used the entire ICU stay to generate our predictive models. Other predictive models, such as SAPS II, use only the first 24 hours of data as prediction at that point of the hospitalization is more clinically useful. However, our objective was to demonstrate how our approach could be used with a clinically significant outcome. Future work could create additional phenotypes using only the first 24 hours of data to generate models. A second limitation is that some of the phenotypes generated are not obvious to clinicians. For example, the main medications in the “anemia” phenotype are diabetic medications. This is likely because non-pharmacologic therapy is the main treatment for anemia and diabetic patients were highly represented in the “anemia” population.

With refinement, future applications of our proposed computational phenotyping method include clinical decision support to quickly identify subgroups of patients at different levels of important clinical outcomes (e.g., mortality, clinical decompensation, hospital readmission, etc.). It could also be used in cohort identification for quality improvement or research projects to find those who share similar characteristics by representing patients’ heterogeneous medical records into membership of phenotypes. In addition, the phenotypes we derived can provide genomic scientists an insight into genotype-phenotype mapping for precision medicine^52, 53^. In conclusion, computational phenotyping using non-negative tensor factorization shows promise as an objective method for identification of important cohorts with promise for clinical, quality improvement and research purposes.

## Electronic supplementary material

Supplementary materials
