# Supplementary material for: Discriminative and Distinct Phenotyping by Constrained Tensor Factorization
Source: Sci Rep. 2017 Apr 25;7:1114. doi: 10.1038/s41598-017-01139-y (PMC5430728; doi:10.1038/s41598-017-01139-y)
Supplement: Supplementary file 1 — Supplementary materials [file 41598_2017_1139_MOESM1_ESM.pdf]

Supplementary materials for Discriminative and Distinct Phenotyping by Constrained Tensor Factorization

Yejin Kim[1], Robert El-Kareh[2], Jimeng Sun[3], Hwanjo Yu[4,*], and Xiaoqian Jiang[2,*]

[1]Department of Creative IT Engineering, Pohang University of Science and Technology, Pohang, Korea
[2]Department of Biomedical Informatics, UC San Diego, La Jolla, CA
[3]School of Computational Science and Engineering, Georgia Institute of Technology, Atlanta, GA
[4]Department of Computer Science and Engineering, Pohang University of Science and Technology, Pohang, Korea
[*]x1jiang@ucsd.edu, hwanjoyu@postech.ac.kr

ABSTRACT

Adoption of Electronic Health Record (EHR) systems has led to collection of massive healthcare data, which creates opportunities and challenges to study them. Computational phenotyping offers a promising way to convert the sparse and complex data into meaningful concepts that are interpretable to healthcare givers to make use of them. We propose a novel supervised nonnegative tensor factorization methodology that derives discriminative and distinct phenotypes. We represented co-occurrence of diagnoses and prescriptions in EHRs as a third-order tensor, and decomposed it using the CP algorithm. We evaluated discriminative power of our models with an Intensive Care Unit database (MIMIC-III) and demonstrated superior performance than state-of-the-art ICU mortality calculators (e.g., APACHE II, SAPS II). Example of the resulted phenotypes are sepsis with acute kidney injury, cardiac surgery, anemia, respiratory failure, heart failure, cardiac arrest, metastatic cancer (requiring ICU), end-stage dementia (requiring ICU and transitioned to comfort-care), intraabdominal conditions, and alcohol abuse/withdrawal.

Supplementary figure

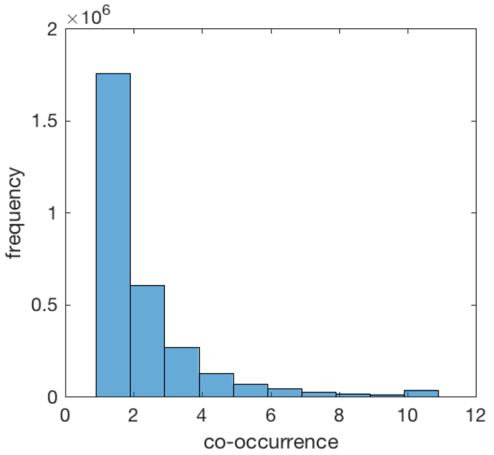

Figure S1. Co-occurence distribution between prescriptions and diagnoses.

Supplementary Tables

Table S1. Time complexity to update $\mathbf{A}_{i:}$ (Eq. 6). Total time complexity for $\mathbf{A}_{i:}$ is bounded by $O(JKR^2)$. Time complexity for $\mathbf{A}$ is $O(JKR^2|L|) + O(JKR^2(I - |L|)) = O(IJKR^2)$.The supervised term had negligible effects on the total time complexity.

	Without supervised term	Supervised term
Compute $\nabla f(\mathbf{A}_{i:})$	$O(JKR^2)$	$O(R)$
Compute $\nabla^2 f(\mathbf{A}_{i:})$	$O((J+K)R^2)$	$O(R^2)$
Inversion $\nabla^2 f(\mathbf{A}_{i:})$	$O(R^3)$	–

Table S2. Time complexity to update $\mathbf{B}$ (Eq. 16). Total time complexity for $\mathbf{B}$ is bounded by $O(IJKR) + O(J^3R^3)$. The similarity-constraint term had negligible effects on the total time complexity.

	Without supervised term	Supervised term
Compute $\nabla g(\mathbf{B})$	$O(IJKR)$	$O(J^2R)$
Compute $\nabla^2 g(\mathbf{B})$	$O((I+K+J^2)R^2)$	$O(J^2R^2)$
Inversion $\nabla^2 g(\mathbf{B})$	$O(J^3R^3)$	–

Table S3. Cross validation. Supervised = the supervised phenotyping for discriminative power, Sim.-based = the similarity-based phenotyping for distinct power, Supervised + Sim.-based = the final model that incorporates the both supervised and similarity-based phenotyping. The models that contain supervision or label information (i.e., likelihood term in supervised phenotyping, discrimination evaluation) used training or test sets.

Model	Used dataset
Constrained tensor factorization	
Supervised	Training set for likelihood term
Sim.-based	Both
Supervised + Sim.-based	Training set for likelihood term
Evaluation	
Discriminative power – Logistic regression parameter	Training set
Discriminative power – AUC, sensitivity, specificity	Test set
Distinctive power	Both

Supplementary methods

Tensor

Tensor is a generalization of matrix. *Order* of a tensor is the number of dimension. A first-order tensor is a vector, a second-order tensor is a matrix, and tensors of order three or higher are called high-order tensors such as (Fig. S2a). *Matricization* is a process of reshaping the tensor into a matrix by unfolding elements of the tensor. Mode-n matricization of a third-order tensor is matricization on each mode n (n = 1, 2, 3) and denoted as $\mathbf{O}_{(n)}$. For example, a third-order tensor $\mathcal{O} \in \mathbb{R}^{2\times2\times2}$ in Fig. S2b and S2c is unfolded on each mode n =1, 2, and 3.

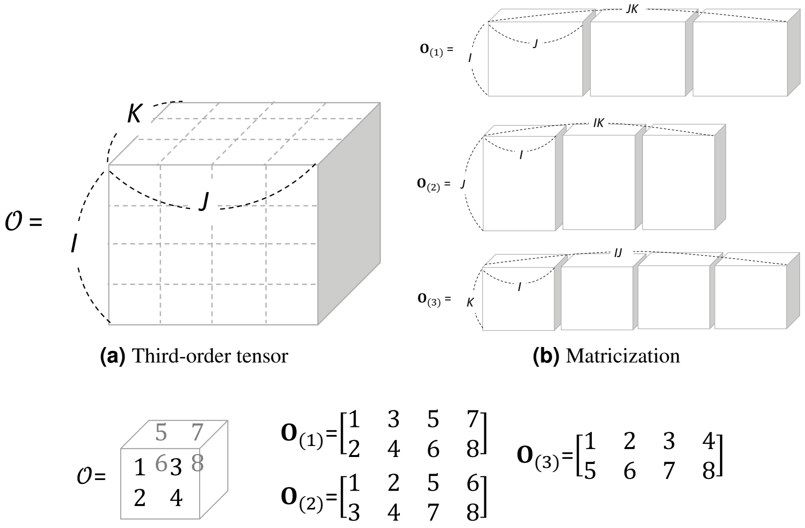

(a) Third-order tensor **(b)** Matricization

(c) Matricization example

Figure S2. A third-order tensor and matricization

A third-order tensor $\mathcal{O} \in \mathbb{R}^{I\times J\times K}$ is *rank-one* if it is an outer product of three vectors $\mathbf{a}, \mathbf{b}$ and $\mathbf{c}$, i.e., $\mathcal{O} = \mathbf{a} \circ \mathbf{b} \circ \mathbf{c}$ where $\circ$ means the vector outer product (Fig. S3). $\mathcal{O}_{ijk}$ the element at (i, j, k) in the tensor $\mathcal{O}$, is computed as a product of elements in the vectors, i.e., $\mathcal{O}_{ijk} = a_i b_j c_k$.

Nonnegative tensor factorization (NTF)

Tensor factorization

Tensor factorization is a dimensionality reduction approach that represents the original tensor as a lower dimensional latent matrix. The CANDECOMP/PARAFAC (CP)[1,2] model (i.e., CP decomposition) is the most popular tensor factorization method that approximates the original tensor $\mathcal{O}$ as a linear combination of rank-one tensors. That is, a third-order tensor $\mathcal{O}$ is decomposed as

$$\mathcal{O} \approx \sum_{r=1}^{R} \mathbf{A}_{:r} \circ \mathbf{B}_{:r} \circ \mathbf{C}_{:r} \tag{1}$$

where R is a positive integer, and $\mathbf{A}_{:r}, \mathbf{B}_{:r}, \mathbf{C}_{:r}$ is a rth column vector in matrix $\mathbf{A} \in \mathbb{R}^{I\times R}, \mathbf{B} \in \mathbb{R}^{J\times R}$ and $\mathbf{C} \in \mathbb{R}^{K\times R}$, respectively (Fig. S4). Here, the matrices $\mathbf{A}, \mathbf{B}$ and $\mathbf{C}$ are called as *factor matrices*. The number R of rank-one tensors is called as *rank*, and $\mathcal{O}$ is called as a rank-R tensor.

Using the factor matrices, the matricized tensor on each mode is written as

$$\begin{aligned} \mathbf{O}_{(1)} &\approx \mathbf{A}(\mathbf{C} \odot \mathbf{B})^T, \\ \mathbf{O}_{(2)} &\approx \mathbf{B}(\mathbf{C} \odot \mathbf{A})^T, \\ \mathbf{O}_{(3)} &\approx \mathbf{C}(\mathbf{B} \odot \mathbf{A})^T \end{aligned} \tag{2}$$

where $\odot$ is Kharti-Rao product. The CP decomposition in Eq. (1) is also expressed as a normalized form by setting the size of

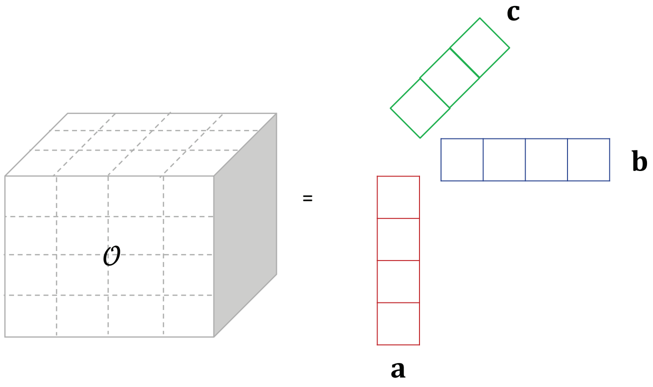

Figure S3. A rank-one tensor $\mathscr{O} = \mathbf{a} \circ \mathbf{b} \circ \mathbf{c}$

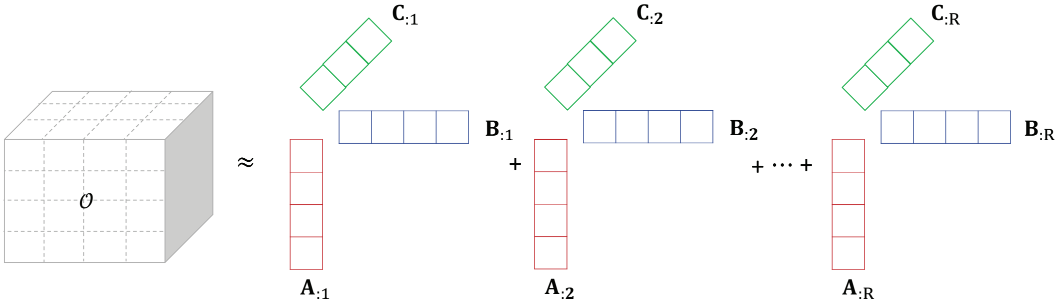

Figure S4. A rank-R tensor $\mathscr{O} \approx \sum_{r=1}^{R} \mathbf{A}_{:r} \circ \mathbf{B}_{:r} \circ \mathbf{C}_{:r}$. $\mathscr{O}$ is approximated as sum of outer product of three column vectors.

column vectors to one and absorbing the weights into λ_r:

$$\mathscr{O} \approx \sum_{r=1}^{R} \lambda_r \overline{\mathbf{A}}_{:r} \circ \overline{\mathbf{B}}_{:r} \circ \overline{\mathbf{C}}_{:r} \tag{3}$$

where $\overline{\mathbf{A}}_{:r} = \frac{\mathbf{A}_{:r}}{||\mathbf{A}_{:r}||_F}$ (same for $\overline{\mathbf{B}}_{:r}, \overline{\mathbf{C}}_{:r}$), and $\lambda_r = ||\mathbf{A}_{:r}||_F ||\mathbf{B}_{:r}||_F ||\mathbf{C}_{:r}||_F$, which is the product of vectors' Frobenius norm.

Nonnegativity

In many real-world applications, tensor factorization is used for analyzing nonnegative data such as count, time and grayscale images. In this case, decomposition into nonnegative factor matrices $\mathbf{A}, \mathbf{B}, \mathbf{C} \geq 0$ is more beneficial than decomposition into matrices containing negative values due to interpretability. When a factor matrix has negative values, and observed data is decomposed into combination of both positive and negative values in the factor matrix, we cannot easily interpret meaning of the negative value in the factor matrices, whereas the positive value indicates the presence of a feature; the zero value indicates the absence.

Optimization

To compute CP decomposition with the nonnegativity constraint, an objective function is to find a tensor $\mathscr{X}$ that best approximates the observed tensor $\mathscr{O}$:

$$\begin{aligned} &\mathscr{X} = \operatorname{argmin} ||\mathscr{O} - \mathscr{X}||_F^2 \\ \text{s.t. } &\mathscr{X} = \sum_{r=1}^{R} \mathbf{A}_{:r} \circ \mathbf{B}_{:r} \circ \mathbf{C}_{:r} \\ &\mathbf{A}, \mathbf{B}, \mathbf{C} \geq 0. \end{aligned} \tag{4}$$

Then, we can rewrite the optimization problem in Eq.(4) with respect to each factor matrix while fixing the other modes:

$$\begin{aligned}
\mathbf{A} &= \text{argmin}_{\mathbf{A}\geq 0}||\mathbf{A}(\mathbf{C}\odot\mathbf{B})^T - \mathbf{O}_{(1)}||_F^2, \\
\mathbf{B} &= \text{argmin}_{\mathbf{B}\geq 0}||\mathbf{B}(\mathbf{C}\odot\mathbf{A})^T - \mathbf{O}_{(2)}||_F^2, \\
\mathbf{C} &= \text{argmin}_{\mathbf{C}\geq 0}||\mathbf{C}(\mathbf{B}\odot\mathbf{A})^T - \mathbf{O}_{(3)}||_F^2.
\end{aligned} \tag{5}$$

References

1. Carroll, J. D. & Chang, J.-J. Analysis of individual differences in multidimensional scaling via an n-way generalization of "eckart-young" decomposition. *Psychometrika* **35**, 283–319 (1970).

2. Harshman, R. A. Foundations of the parafac procedure: Models and conditions for an" explanatory" multi-modal factor analysis (1970).
